# Phytofabrication of ZIF-8 Using Mangrove Metabolites for Dual Action Against Drug-Resistant Microbes and Breast Cancer Cells

**DOI:** 10.3390/biomimetics10110755

**Published:** 2025-11-08

**Authors:** Srinath Rajeswaran, Mithuna Shaji Kumarikrishna, Aneesh Giriprasath, Kandi Sridhar, Murugan Anbazhagan, Siva Vadivel, Maharshi Bhaswant

**Affiliations:** 1 Department of Biotechnology, Karpagam Academy of Higher Education, Coimbatore 641021, Tamil Nadu, India; mithunask2001@gmail.com (M.S.K.); aneeshgiri103@gmail.com (A.G.); 2 Department of Food Technology, Karpagam Academy of Higher Education, Coimbatore 641021, Tamil Nadu, India; sridhar4647@gmail.com; 3 Department of Science and Humanities, Karpagam College of Engineering, Coimbatore 641032, Tamil Nadu, India; murugan.a@kce.ac.in; 4 Centre for Energy and Environment, Karpagam Academy of Higher Education, Coimbatore 641021, Tamil Nadu, India; 5 Department of Physics, Karpagam Academy of Higher Education, Coimbatore 641021, Tamil Nadu, India; 6 Department of Biotechnology, SRM—Institute of Science and Technology, Tiruchirappalli Campus, Tiruchirappalli 621105, Tamil Nadu, India; 7 New Industry Creation Hatchery Center (NICHe), Tohoku University, Sendai 980-8579, Japan

**Keywords:** Zeolitic Imidazolate Framework-8, *Conocarpus erectus*, drug resistance, antimicrobial, anticancer

## Abstract

Green nanotechnology offers a sustainable and eco-friendly approach for nanoframework synthesis. The present study intended to synthesize a novel eco-friendly encapsulated Zeolitic Imidazolate Framework-8 (ZIF-8) in a one-pot method using metabolites from the mangrove plant *Conocarpus erectus* (CE). Gas Chromatography–Mass Spectrometry (GC-MS) analysis of the extract revealed the presence of important bioactive metabolites. The synthesized material was evaluated by UV-Vis spectroscopy, X-ray diffraction (XRD), particle size analysis (PSA), zeta potential measurement, high-resolution transmission electron microscopy (HR-TEM), and Fourier transform infrared (FT-IR) spectroscopy studies. The environment-friendly mangrove metabolites aided by Zeolitic Imidazolate Framework-8 was found to be crystalline, rhombic dodecahedron structured, and size dispersed without agglomeration. The nanomaterial possessed a broad antimicrobial effect on drug-resistant microorganisms, including *Candida krusei*, *Escherichia coli*, *Streptococcus* Sp., *Staphylococcus aureus*, *Enterococcus* Sp., *Pseudomonas aeruginosa*, *Klebsiella pneumoniae*, *C. propicalis*, and *C. albicans*. Further, its cytotoxicity against MDA-MB-231 cells was found to be efficient. The morphological alterations exhibited by the antiproliferative impact on the breast cancer cell line were detected using DAPI and AO/EB staining. Therefore, ZIF-8 encapsulated mangrove metabolites could serve as an effective biomaterial with biomedical properties in the future.

## 1. Introduction

Plant extracts are widely used to synthesize nanomaterials, which have a number of benefits, including improved stability, biocompatibility, and economical manufacture. Recently, mangrove ecosystems have attracted increasing attention as sustainable sources for the synthesis of green nanomaterials for biomedical and environmental applications. Mangrove based nanomaterials exhibit unique physicochemical properties, including high porosity with high surface area and the ability to form stable complexes with several organic and inorganic compounds [1]. One of the two species in the *Conocarpus* genus, *Conocarpus erectus,* is a member of the *Combretaceae* family rich in phytochemicals used as folklore medicine for fever, diabetes, syphilis, gonorrhea, anemia, orchitis, swellings, and diarrhea [2,3]. *Conocarpus* species’ bioactive molecules can be utilized for pharmacological applications and as functional foods. *Conocarpus* spp. are found to comprise vanillic acid, p-coumaric acid, quercetin, rutin hydrate, flavone, t-ferulic acid, sinapic acid, and protocatechuic acid [4].

Multidrug resistance (MDR) is the increasing prevalence and growing concern due to the limited development of novel and effective antimicrobials. Antimicrobial resistance (AMR) in bacteria has evolved more quickly as a result of the extensive use of antibiotics, posing a significant and growing threat to global public health [5]. In recent decades, oncology has undergone a major revolution with the introduction of increasingly personalized treatments. Cancer has been the major cause of disease-related death for decades and poses a serious threat to human health. With the fast development of nanomedicine, nanoscale metal–organic frameworks are increasingly recognized for their potential applications in biomedical imaging and cancer therapy [6]. To enhance current therapeutic strategies and discover new potential treatments for these diseases, novel drug delivery carriers must be developed. In recent decades, nanoscaled metal–organic frameworks have become a new class of organic–inorganic hybrid coordination polymeric nanomaterials that have shown considerable promise in the biomedical field. Zeolitic Imidazolate Frameworks are formed by coordinating imidazole or imidazole derivatives with tetrahedral transition metal ions, with imidazolate units serving as the linkers that bind the metal ions together. Due to their high drug loading and inherent biodegradability, they have garnered a lot of interest as pH-sensitive drug carriers [7,8]. ZIF-8 is a highly important material with a sodalite structure, with small pore apertures of around 3.4 Å and interior cavities measuring about 11.6 Å, restricting the entry of bigger molecules. It has exceptional thermal and chemical stability, maintaining structural integrity after seven days of incubation at 50 °C in both aqueous and organic mediums. Its porosity allows for the encapsulation of secondary metabolites from plant extracts, making it a promising option for targeted drug delivery applications [9]. ZIF based materials can be systematically validated by several spectroscopic and microscopic techniques [10]. Zeolitic Imidazolate Framework-8 (ZIF-8) and its related compounds possess significant antibacterial properties due to the controlled release of antimicrobial zinc ions (Zn^2+^) and their highly porous structure, which enhances contact with bacterial cells [11]. ZIF-8 exhibits pH-responsive drug release, releasing pharmaceuticals at a better rate at acidic pH values (5.5–6), mimicking the internal circumstances of cancer cells, specifically in endosomes and lysosomes. This targeted release mechanism has the potential to enhance therapeutic efficacy while minimizing off-target effects [12]. Based on this information, this study aims to synthesize *Conocarpus erectus*-mediated Zeolitic Imidazolate Framework-8 nanoconjugate in a one-pot method using a biosynthesis approach and to evaluate their antimicrobial and anticancer activities.

## 2. Materials and Methods

### 2.1. Plant Material and Extraction

The plant *Conocarpus erectus* leaves were collected at Dharapuram, Tamil Nadu, India. After washing, drying, and milling, the *Conocarpus erectus* powder (50 g) performed soxhlation method utilizing methanol solvent at 50 °C for 48 h. Then, the resulting extract was concentrated using a rotary evaporator and used for experiments.

### 2.2. GC-MS Investigation

*C. erectus* methanolic extract was subjected to GC-MS analysis utilizing a Shimadzu GC-2014 (Kyoto, Japan), system fitted with a HP-5MS capillary column (5% phenyl methyl siloxane; 30.0 m × 250 μm × 0.25 μm) and a mass selective detector (MS). Helium was utilized as the carrier gas at a steady flow rate of 1 mL/min. The temperature of the column was first set at 40 °C for 10 min, then increased up to 250 °C at a rate of 3 °C per minute, and then kept isothermally for 5 min. An ionization energy of 70 eV was used in the mass spectrometer’s operation. The resulting mass spectra were compared to those in the NIST spectral collection in order to identify the compound.

### 2.3. Synthesis of ZIF-8 and CE@ZIF-8

The synthesis of ZIF-8 was performed [9,11] and to synthesize CE@ZIF-8 in the one-pot method, two distinct solutions were synthesized with modifications. The first step involved dissolving 2 g of zinc nitrate hexahydrate in 20 mL of methanol. Further, 40 mL of methanol was used to dissolve 4 g of 2-methylimidazole and 1 g of the *C. erectus* extract, which was then agitated with a magnetic stirrer for the entire night at 400 rpm. The *C. erectus* extract and 2-methylimidazole solution were agitated for an hour before the zinc nitrate solution was added dropwise. A 24 h rest period at room temperature was then given to the combined mixture. The produced CE@ZIF-8 was then centrifuged for 15 min at 10,000 rpm. The product was then dried at 70 °C after being cleaned three times with methanol. Further, CE@ZIF-8 material was ground into a powder using a mortar and pestle [9,10,11].

### 2.4. Characterization Techniques

The instruments including Shimadzu UV-1780 (Kyoto, Japan), Shimadzu IR Tracer-100 spectrophotometer (Kyoto, Japan), Bruker D8 advanced ECO X-ray diffractometer (Karlsruhe, Germany), JEOL JEM-2100 plus (Tokyo, Japan), and Zetasizer Nano ZS 90 (Malvern Instruments, Malvern, UK) were utilized for the characterization of the material.

### 2.5. Antimicrobial Activity

#### 2.5.1. Microbial Strains

The drug-resistant pathogen strains *Escherichia coli*, *Staphylococcus aureus*, *Streptococcus* Sp., *Enterococcus* Sp., *Pseudomonas aeruginosa*, and *Klebsiella pneumoniae* were experimented with for the study. Similarly, *Candida krusei*, *C. propicalis*, and *C. albicans* were procured from Bioline laboratory, Coimbatore, India.

#### 2.5.2. Minimum Inhibitory Concentration (MIC) Determination

Different concentrations were aliquoted from the stock (10 mg/mL), ranging from 7.8 μg to 250 μg/mL. Each test tube was filled with 500 µL of bacterial culture (standardized using CFU) after 0.5 mL of the test dilution was added to 2 mL of nutrient broth (NB). NB was the control. The control and test sample NB tubes were placed at 37 °C for 24 h. The bacterial growth was evaluated at A_600_ after incubation using a spectrophotometer. The conventional microdilution technique was performed [13]. Likewise, the MIC analysis of fungal strains was carried out in accordance with Arsène et al., 2023 [14].

#### 2.5.3. Antimicrobial Assay

Fresh bacterial cultures were standardized to a dose of 1 × 10^7^ CFU/mL based on optical density measurements. Using sterile cotton swabs, the standardized inoculum was equally distributed across Mueller–Hinton Agar (MHA) plates to create uniform microbial lawns. The extract was poured onto sterile 5 mm discs which were then positioned on the plates at doses of 75 μg/mL and 150 μg/mL [15]. Streptomycin served as the positive control. Zones of inhibition (in mm) were measured after 24 h of incubation at 37 °C to assess antibacterial activity. The antifungal evaluation was performed with few modifications. *Candida* species were cultured in potato dextrose broth at 35 °C for 48 hrs. Further the standardized dose of 1 × 10^6^ CFU/mL of the fungal cells were spread onto freshly prepared potato dextrose agar (PDA) plates. Discs impregnated with the sample at dosages of 100 μg/mL and 200 μg/mL were placed onto the inoculated plates. Amphotericin B was used as the standard antifungal agent. Inhibition zones were recorded after appropriate incubation [16].

### 2.6. Cell Lines and Culture Conditions

The National Centre for Cell Sciences (NCCS) in Pune, India, provided the human breast cancer cell line MDA-MB-231. Following NCCS recommendations, cells were cultivated in Dulbecco’s Modified Eagle Medium (DMEM) supplemented with 10% fetal bovine serum (FBS) and 1% penicillin-streptomycin. They were then kept in a humidified incubator at 37 °C with 5% CO_2_.

#### 2.6.1. Cytotoxicity Valuation Assay

Cancer cells were individually seeded onto 96-well plates at a density of 1 × 10^4^ cells per well. After that, the cells were incubated overnight at 37 °C in a humidified atmosphere with 5% CO_2_. Then, cells were exposed to a range of CE@ZIF-8 doses, from 15 μg/mL to 120 μg/mL. The culture media was replaced after the incubation period and in each well was added with 20 μL of MTT ((4,5-dimethylthiazol-2-yl)-2,5-diphenyl tetrazolium bromide) solution (5 mg/mL in PBS). Then, the plates were incubated in the dark for three hours. After aspirating the MTT solution, the formazan crystals were dissolved using 200 μL of DMSO. A microplate reader was used to measure the absorbance at 570 nm, and the resulting percentage of cell viability was analyzed [17].

#### 2.6.2. Morphological Study

Cells were seeded at a concentration of 5 × 10^4^ cells/mL in 6-well plates and incubated in a conventional environment with 37 °C, 5% CO_2_, and a humidified atmosphere. After incubation, cells were treated with CE@ZIF-8 concentrations. Following treatment, cells were fixed with (3:1) ethanol:acetic acid solution. After that, morphological alterations were noted at 40× magnification using a bright-field microscope (Labored TCM 400, Gurgaon, India) [18].

#### 2.6.3. Acridine Orange/Ethidium Bromide (AO/EB) Staining

On sterile coverslips set inside 6-well plates, cancer cells were seeded at a concentration of 5 × 10^5^ cells per well. Cells were either left untreated or exposed to the CE@ZIF-8 IC_50_ concentration. Acridine orange/ethidium bromide (AO/EB) solution (50 μL of 5 mg/mL) was used to stain the cells after treatment, and they were then incubated for 30 min under the same culture conditions. To remove the extra stain, the coverslips were then gently rinsed with 1× phosphate-buffered saline (PBS). The EVOS^®^ FLoid^®^ Cell Imaging System (Life Technologies, Carlsbad, CA, USA) with a 20× objective lens was used to view cellular morphology.

#### 2.6.4. DAPI Staining

For DAPI (4′,6-diamidino-2-phenylindole) staining, cells were seeded into 6-well plates and after incubation, cells were exposed to the corresponding IC_50_ concentration. After treatment, cells were stained with DAPI (2 μg/mL) and then incubated for 30 min. The extra stain was removed by 1× phosphate-buffered saline (PBS). Finally, the EVOS^®^ FLoid^®^ Cell Imaging Station was used to visualize nuclear morphology.

### 2.7. Assessment of Toxicity Using Artemia salina

Using the brine shrimp *Artemia salina* lethality test, the biosafety of the produced CE@ZIF-8 was assessed [19]. The OECD criteria and procedures for medicines and nanomaterials were followed during the experimental process [20]. *A. salina* nauplii that had just hatched were experimented on a 12-well plate and left in seawater for 24 h with varying doses of CE@ZIF-8 (25–200 μg/mL). For comparative research, a well that contains *A. salina* nauplii without samples is regarded as a negative control. Cisplatin, a marketed anticancer medication, served as a positive control.

### 2.8. Statistical Analysis

GraphPad Prism version 9.0 was used to analyze one-way analysis of variance and statistical analysis was carried out using Microsoft Excel. The data was presented with mean ± standard deviation (SD) after analysis.

## 3. Results and Discussion

### 3.1. Metabolite Analysis

**Figure 1:**
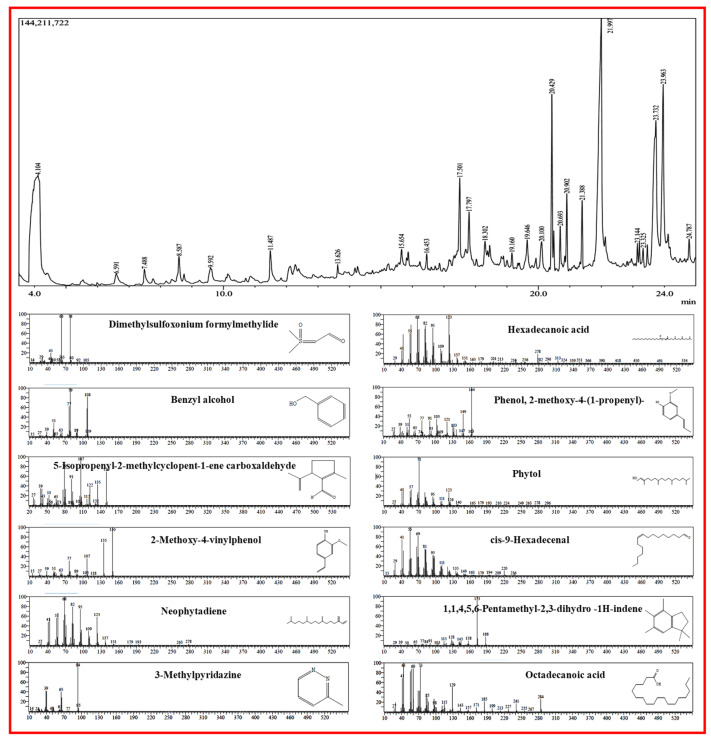
GC and mass spectrometric representation of metabolites from *C. erectus* extract.

In Figure 1, the distinctive chromatogram signals are depicted during their RT, and Table 1 lists the compounds that were captured. In the GC chromatogram, *C. erectus* methanol extracts displayed 25 peaks. Finding the active compounds and analyzing GC-MS was carried out using the Wiley library and National Institute of Standards and Technology (NIST) database. Among the compounds identified in GC-MS are Dimethylsulfoxonium formylmethylide, Benzyl alcohol, 2-Methoxy-4-vinylphenol, Phenol, 1,1,4,5,6-Pentamethyl-2,3-dihydro-1H-indene, 5-Isopropenyl-2-methylcyclopent-1-ene carboxaldehyde, 1-Dodecanol3 7 11-trimethyl, 5,5,8a-Trimethyl-3,5,6,7,8,8a-hexahydro-2H-chromen, Neophytadiene, Hexadecanoic acid, Phytol, cis-9-Hexadecenal, and Octadecanoic acid which were regarded as antimicrobial agents. Dimethylsulfoxonium formylmethylide has antimicrobial properties [21], Benzyl alcohol is an aromatic alcohol used as an antimicrobial preservative at optimum concentrations [22], 2-Methoxy-4-vinylphenol have many biological activities [23], Phenols are a natural methoxyphenol compound that has antimicrobial activity against foodborne pathogens [24], 1,1,4,5,6-Pentamethyl-2,3-dihydro -1H-indene have antifungal properties [25], 5-Isopropenyl-2-methylcyclopent-1-ene carboxaldehyde have antimicrobial activity [26], and Neophytadiene [27], Hexadecanoic acid [28], Phytol, cis-9-Hexadecenal, and Octadecanoic acid possess antimicrobial actions [29].

### 3.2. Synthesis and Characterization of CE@ZIF-8

**Figure 2:**
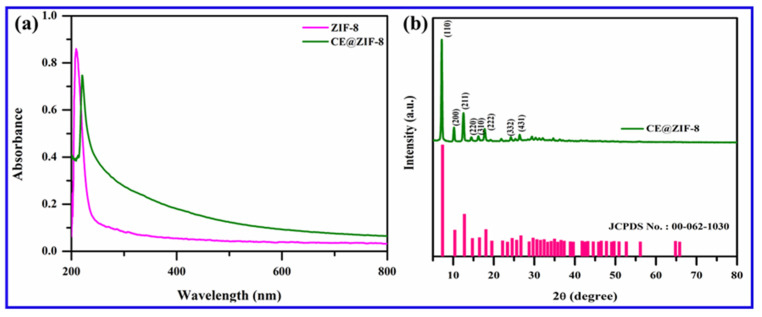
(**a**) UV spectrum of ZIF-8 and CE@ZIF-8 and (**b**) XRD pattern of CE@ZIF-8.

**Figure 3:**
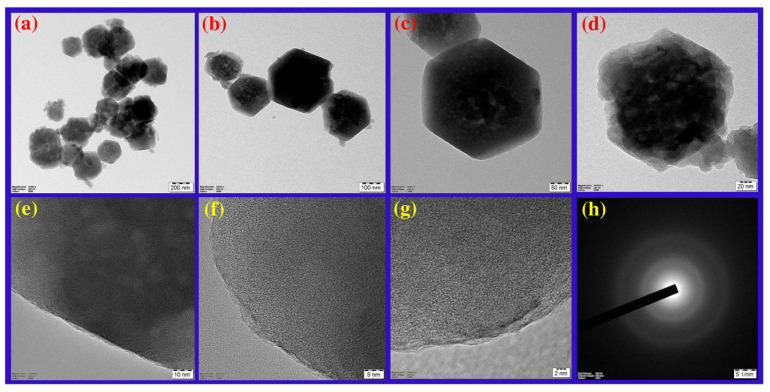
HR-TEM observation of CE@ZIF-8 at (**a**) 200 nm, (**b**) 100 nm, (**c**) 50 nm, (**d**) 20 nm, (**e**) 10 nm, (**f**) 5 nm, (**g**) 2 nm, and (**h**) SAED pattern.

**Figure 4:**
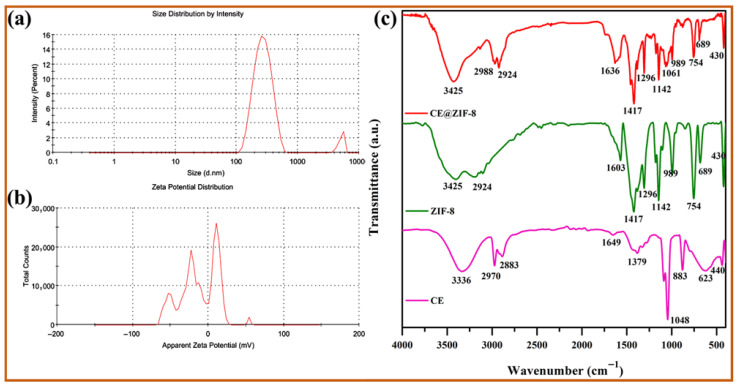
(**a**) Particle size distribution, (**b**) zeta potential analysis, and (**c**) FT-IR spectra of CE extract, ZIF-8, and CE@ZIF-8.

ZIF-8 is formed by the simple reaction of metal ions and organic linkers in a solution, resulting in a porous coordination framework. Our study shows that *Conocarpus erectus* extract was successfully encapsulated on ZIF-8 framework, forming CE@ZIF-8. The bioactive molecules present in the CE, which possesses therapeutic properties, are selectively incorporated into the ZIF-8 structure during the in situ synthesis process. This encapsulation is achieved by including the CE during the self-constructing of the metal ions and organic linkers, allowing the bioactive molecules to become trapped within the porous ZIF-8 lattice as it forms. At 209 nm and 220 nm, the ZIF-8 and CE@ZIF-8 formation showed an absorbance peak (Figure 2a). The obtained results were comparable with the reports of Arya et al., 2023, and Raju et al., 2023 [9,10]. The crystalline nature of the prepared material is shown in Figure 2b. The CE@ZIF-8 XRD peaks at 2θ = 7.21°, 10.25°, 12.52°, 14.53°, 16.29°, 24.36°, and 26.49° are the planes that correspond to (110), (200), (211), (220), (310), (222), (332), and (431). The CE@ZIF-8 XRD was affirmed with JCPDS File No.00-062-1030. The yielded results were comparable with the previously stated reports [9,11]. The size-dispersed range of CE@ZIF-8 is observed between 150 and 350 nm. The CE@ZIF-8 in HR-TEM possessed smooth surfaces with rhombic dodecahedron shape (Figure 3a–h). The Dynamic Light Scattering (DLS) analysis was performed to determine the hydrodynamic particle size and colloidal stability, which served to corroborate the morphological data obtained from HR-TEM. The results can be compared to those of biosynthesized ZIF-8 material [9]. The CE@ZIF-8 possessed a polydispersity index (PDI) of 0.32, which is higher than ZIF-8 [11] with a Z-average (d. nm) of 272.8, indicating CE@ZIF-8 is stable and the regular distribution curve is shown in Figure 4a. The quantity of electrostatic potential on the exterior of CE@ZIF-8 was recorded as −11.9 mV (Figure 4b). The formed CE@ZIF-8 exhibited negative zeta potential. This negative surface charge is a key factor in its enhanced bactericidal activity. This study shows that the CE@ZIF-8 exhibits suggestively greater antibacterial properties. This improved efficacy, combined with the nanocomposite’s optimal particle size and negative surface charge, makes it a promising candidate for various pharmaceutical formulations, as it demonstrates potent antibacterial effects with low toxicity [30]. The FT-IR results (Figure 4c) depicted the vibrational profile of various atomic and polar bonds in the *C. erectus* plant extract, ZIF-8 and CE@ZIF-8 was examined in the range between 4000 cm^−1^ and 500 cm^−1^. The *C. erectus* plant extract showed a significant vibrational pattern at 2970 cm^−1^, 2883 cm^−1^, 1649 cm^−1^, 1379 cm^−1^, and 1048 cm^−1^. The CE@ZIF-8 depicted major peak range from the plant extract sourced functional groups at 2988 cm^−1^, 2924 cm^−1^, 1636 cm^−1^, 1142 cm^−1^, and 1061 cm^−1^ which majorly consisted of the presence of aromatic, amines, and alkenes functional groups with C–N stretching and C–H stretch.

### 3.3. Microbicidal Activity

**Figure 5:**
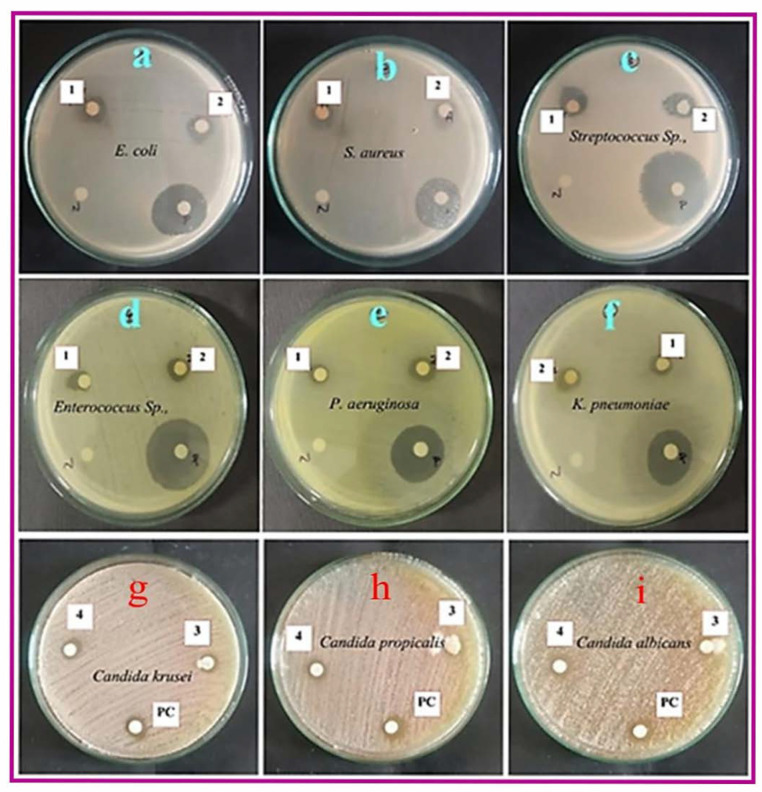
Antibacterial (**a**–**f**) and anti-candidal activity (**g**–**i**) of CE@ZIF-8 (1—75 μg/mL, 2—150 μg/mL, 3—100 μg/mL, 4—200 μg/mL; N—negative control; P/PC—positive control).

The antibacterial activity of CE@ZIF-8 was evaluated over a concentration range of 7.8–250 µg/mL. The dosages and results are demonstrated in Table 2, Table 3, Table 4 and Table 5. After evaluating the potential of *C. erectus* extract and ZIF-8 (data not given) against bacterial pathogens, the bactericidal activity of CE@ZIF-8 (Figure 5) was experimented. The antibacterial activity of CE@ZIF-8 was shown to be minimal against *S. aureus* (7.6 mm) and maximal against *Streptococcus* Sp. (12.2 mm) at 150 μg/mL dosage. For fungicidal activity, maximum anti-candidal activity of CE@ZIF-8 was shown at 200 μg/mL dosage towards *C. krusei* (7.2 mm) and minimum activity against *C. propicalis* was noticed. The antimicrobial mechanism of CE@ZIF-8 may be multifaceted, involving a series of destructive cellular events. Furthermore, the material can be internalized by the cell, where it induces genotoxicity by breaking down DNA and disrupts essential metabolic functions by interacting with the thiol groups of key proteins. Previous research has demonstrated the antibacterial effectiveness of Ag@ZIF-8@GO and Ag-Cu@ZIF-8@GO against both Gram-positive (*S. aureus*) and Gram-negative (*E. coli*) bacteria [31]. The presence of zinc ions and enclosed bioactive chemicals within CE@ZIF-8’s structural framework is what gives it its antibacterial activity. As a zinc ion and antimicrobial agent reservoir, CE@ZIF-8 has antibacterial properties similar to those of metal nanoparticles but different from traditional antibiotics in terms of mechanism. By breaking down the bacterial cell wall, the antibacterial process causes the cytoplasmic contents to flow out. Additionally, cellular internalization, DNA fragmentation, ion channel and membrane disruption, and interactions with protein thiol groups are some of the ways that the metal–organic framework and its derivatives support antibacterial activity [32]. The primary mechanism underlying the antibacterial activity of MOFs is likely the bulk release of metal ions, either as free cations or as fragments of the MOF structure. The encapsulation capability of metal–organic frameworks (MOFs) enable the incorporation of therapeutic agents or bioactive molecules within their porous structure [33]. The extent of antimicrobial activity is closely correlated with the structural rigidity (or ‘hardness’) of the MOF, which influences the ease of cation release. Increased ion release is associated with enhanced antibacterial efficacy. Our study supports the hypothesis that the microbicidal activity of CE@ZIF-8 is predominantly enhanced by the delivery of zinc ions. Additionally, the organic linkers within the MOF also contribute to the observed antimicrobial effects.

### 3.4. Cytotoxic Efficacy and Morphological Assessment

**Figure 6:**
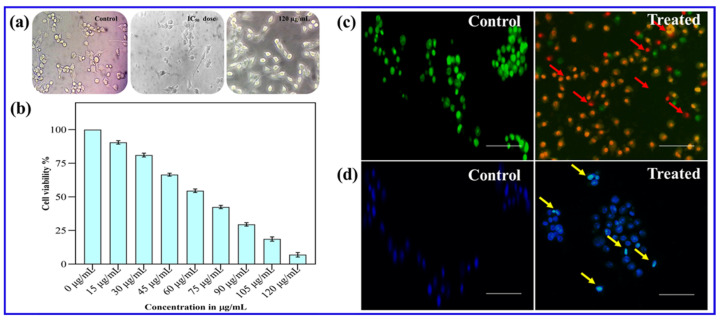
(**a**) Phase-contrast microscopic observation of CE@ZIF-8 against MDA-MB-231 cells. (**b**) Cytotoxicity testing using MTT, (**c**) morphological analysis using AO/EB staining, and (**d**) DAPI staining were performed on cancer cells treated with IC_50_ concentrations of CE@ZIF-8 (scale—100 µm, red and yellow arrows- apoptosis induction).

The cytotoxic effects of CE@ZIF-8 on MDA-MB-231 breast cancer cells were evaluated using MTT assay. CE@ZIF-8 confirmed noteworthy cytotoxicity against MDA-MB-231 breast cancer cells (Figure 6b, Table 6). CE@ZIF-8 inhibited cell growth with a strong IC_50_ value of 60.0 ± 0.5 µg/mL after 24 h. The nanomaterial concentration demonstrated a direct correlation with intracellular reactive oxygen species (ROS) generation within cancer cells. Phase-contrast microscopy (Figure 6a) revealed significant morphological changes in the cancer cells following treatment with CE@ZIF-8. As shown in Figure 6b, treated cells exhibited a notable decrease in cell density. These observed morphological changes, such as reduced cell density and the distinctive features of cell rounding and shrinkage, strongly suggest that CE@ZIF-8 induces apoptosis in MDA-MB-231 cancer cells. Most chemotherapeutic agents induce cell death either through direct nucleic acid damage or by disrupting the cellular redox homeostasis, with reactive oxygen species (ROS) serving as a central mediator of apoptosis in both mechanisms [34]. To date, there have been no reported studies on the encapsulation of *C. erectus* extract within ZIF structures for biomedical applications. This study demonstrates the successful encapsulation of *C. erectus* extract within ZIF-8 and highlights its potential in breast cancer therapy as well as in combating infectious agents. The synergistic interaction between the phytoconstituents of *C. erectus* and the zinc ions released from ZIF-8 facilitates enhanced reactive oxygen species (ROS) generation, important to nuclear damage and ultimately promoting cancer cell death.

### 3.5. Fluorescent Staining Studies

The anticancer efficacy of chemotherapeutic agents is primarily attributed to the group of reactive oxygen species, which induce nuclear damage and activate cell death pathways. Current studies have demonstrated that nanomaterial-treated cells exhibit a ROS burst, resulting in protein oxidation, DNA fragmentation, and lipid peroxidation—collectively leading to apoptosis [35,36]. Antitumor drugs can be selectively internalized by cancer cells and induce cell death through the apoptotic pathway [37]. In this context, the synthesized CE@ZIF-8 effectively induced apoptosis in cancer cells with significantly low concentrations. To evaluate this effect, the CE@ZIF-8 treated cancer cells were stained with acridine orange and ethidium bromide (AO/EB). Post-treatment, the cancer cells exhibited morphological features of apoptosis, including membrane disintegration, allowing ethidium bromide to penetrate and intercalate with DNA, resulting in a red fluorescence that masked the green fluorescence of acridine orange. This color shift, as shown in Figure 6c, confirms apoptosis induction by CE@ZIF-8. DAPI staining was accomplished to confirm nuclear damage and apoptosis induction (Figure 6d). Control cells displayed light blue fluorescence, indicative of intact nuclei and cytoplasm, whereas cells treated with CE@ZIF-8 showed intense blue fluorescence, suggesting nuclear fragmentation. Moreover, CE@ZIF-8-treated cells exhibited distinct cytological changes, including nuclear shrinkage, micronuclei formation, binucleation, and chromatin condensation, confirming apoptotic cell death. These findings indicate that CE@ZIF-8 induce ROS-mediated nuclear damage in cancer cells, leading to apoptosis. Recently, synergistic therapy is widely recognized as a promising cancer treatment approach. Previous reports state that ZIF-8 exhibits noticeable pH-responsive drug release capabilities within the acidic range (pH 5.0–7.4), confirming its suitability for anticancer applications [38,39]. The supporting literature [40] also highlights the use of *C. erectus* methanolic extract for anticancer applications. Therefore, the *C. erectus* extract was utilized in the synthesis of CE@ZIF-8, demonstrating substantial antimicrobial and anticancer potential.

### 3.6. Evaluation of Toxicity Using A. salina

**Figure 7:**
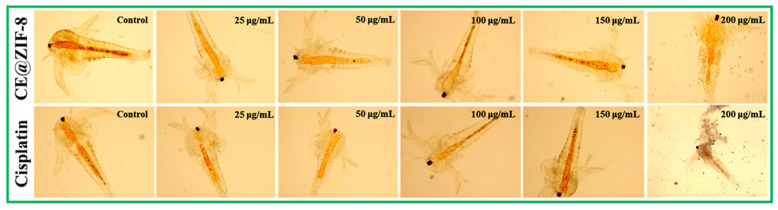
Toxicity assessment of *Artemia salina* nauplii post exposure to CE@ZIF-8 and cisplatin at different concentrations (10× magnification).

According to OECD guidelines for chemical testing, *A. salina* based assays were considered for acute toxicity evaluation, as these organisms exhibit sensitivity comparable to that of higher animals. Figure 7 displayed the findings of toxicity tests of ZIF-8 mediated by *C. erectus* extract and employing *A. salina*. The findings verified that the dosage of CE@ZIF-8 against *A. salina* nauplii was up to 200 μg/mL. At a maximal dose of 250 μg/mL (data not given) and above, CE@ZIF-8 showed little harm, according to the toxicity study. In this instance, groups that received cisplatin demonstrated a notable degree of lethality and behavioral alterations following treatment, which were consistent with assessments that have been published [10,36].

## 4. Conclusions

This study demonstrates a sustainable approach for the eco-friendly synthesis of encapsulating *Conocarpus erectus* extract within Zeolitic Imidazolate Framework-8. The key metabolites including Dimethylsulfoxonium formylmethylide, Benzyl alcohol, Phenols, 5-Isopropenyl-2-methylcyclopent-1-ene carboxaldehyde, 2-Methoxy-4-vinylphenol, Neophytadiene, Hexadecanoic acid, Phytol, cis-9-Hexadecenal, 1,1,4,5,6-Pentamethyl-2,3-dihydro -1H-indene, and Octadecanoic acid were seen as responsible for the experimented biological activities. Moreover, the CE@ZIF-8 exhibited potent antimicrobial efficacy against drug-resistant pathogens. It also demonstrated significant cytotoxic effects against MDA-MB-231 cancer cells at optimum dosages, as evidenced by morphological changes due to triggered apoptosis which was observed through AO/EB and DAPI staining. However, the present study was primarily limited to evaluating only basic encapsulation efficiency and biocompatibility. Therefore, detailed drug release profiling and quantitative loading kinetics will be addressed in future investigations. These preliminary findings suggest that ZIF-8 encapsulated with *C. erectus* metabolites, exhibits promising biomedical potential and could be further developed for antimicrobial and anticancer applications.

## Figures and Tables

**Table 1:** Key chemical constituents from methanolic extract of *Conocarpus erectus*.

Peak	R. Time	Area (%)	Height (%)	Compound Name
1	4.104	25.20	7.25	Dimethylsulfoxonium formylmethylide
2	6.591	1.14	0.74	2-Furancarboxaldehyde, 5-methyl-
3	7.488	0.78	1.04	3-Methylpyridazine
4	8.587	1.12	1.76	Benzyl alcohol
5	9.592	1.27	0.98	Hydrouracil, 1-methyl-
6	11.487	1.72	2.14	Ethanone, 1-(2-methylphenyl)-
7	13.626	0.41	0.85	2-Methoxy-4-vinylphenol
8	15.654	0.55	1.08	Phenol, 2-Methoxy-4-(1-propenyl)-, (Z)-
9	16.453	0.58	1.15	1,1,4,5,6-Pentamethyl-2,3-dihydro-1H-indene
10	17.501	2.84	5.66	Diethyl Phthalate
11	17.797	2.42	3.37	5-Isopropenyl-2-methylcyclopent-1-enecarboxaldehyde
12	18.302	2.22	1.66	1,1-Dimethyl-1,2,3,5,7,8,9,9a-octahydro-benzocyclohepten-6-one
13	19.160	0.42	1.03	1-Dodecanol, 3,7,11-trimethyl-
14	19.646	1.72	2.01	Tetradecanoic acid
15	20.100	1.63	1.94	5,5,8a-Trimethyl-3,5,6,7,8,8a-hexahydro-2H-chromene
16	20.429	6.46	12.58	Neophytadiene
17	20.693	1.10	2.93	Neophytadiene
18	20.902	2.67	5.46	Neophytadiene
19	21.388	2.00	4.77	Hexadecanoic acid, methyl ester
20	21.997	22.53	17.01	n-Hexadecanoic acid
21	23.144	0.51	1.48	9,12-Octadecadienoic acid (Z,Z)-, methyl ester
22	23.325	0.48	1.21	Phytol
23	23.732	12.14	9.17	cis-9-Hexadecenal
24	23.963	7.46	11.27	Octadecanoic acid
25	24.787	0.64	1.45	E-10,13,13-Trimethyl-11-tetradecen-1-ol acetate
Total		100.00	100.00	

**Table 2:** Minimal Inhibitory Concentrations (MIC) of CE@ZIF-8 against pathogenic bacterial strains.

S. No	Name of the Strains	7.8 μg/mL	15.6 μg/mL	31.25 μg/mL	62.5 μg/mL	125.0 μg/mL	250.0 μg/mL	(+) ve C	(−) ve C
1.	*E. coli*.	+++	+++	+++	++	+	*	−−	+++
2.	*S. aureus*	+++	+++	+++	++	+	*	−−	+++
3.	*Streptococcus* Sp.	+++	+++	++	*	−−	−−	−−	+++
4.	*Enterococcus* Sp.	+++	+++	+++	++	+	*	−−	+++
5.	*P. aeruginosa*	+++	+++	++	+	*	−−	−−	+++
6.	*K. pneumoniae*	+++	+++	++	+	*	−−	−−	+++

+++ depicts highly turbid; ++ depicts turbid; + indicates cloudiness; * MIC; −− no growth, C—control.

**Table 3:** Minimal Inhibitory Concentrations (MIC) of CE@ZIF-8 against pathogenic fungal strains.

S. No	Name of the Strains	7.8 μg/mL	15.6 μg/mL	31.25 μg/mL	62.5 μg/mL	125.0 μg/mL	250.0 μg/mL	(+) ve C	(−) ve C
1.	*C. krusei*	+++	+++	++	++	+	*	−−	+++
2.	*C. propicalis*	+++	+++	++	++	+	*	−−	+++
3.	*C. albicans*	+++	+++	++	++	+	*	−−	+++

+++ depicts highly turbid; ++ depicts turbid; + indicates cloudiness; * MIC; −− no growth, C—control.

**Table 4:** Antibacterial activity of CE@ZIF-8.

S. No	Name of the Strains		Zone of Inhibition (mm)		
		75 μg/mL	150 μg/mL	(+) ve C	(−) ve C
1.	*E. coli.*	5.7 ± 0.52	8.1 ± 0.26	18.2 ± 0.15	−−
2.	*S. aureus*	4.5 ± 0.25	7.6 ± 0.45	15.6 ± 0.36	−−
3.	*Streptococcus* Sp.	8.3 ± 0.51	12.2 ± 0.47	24.4 ± 0.43	−−
4.	*Enterococcus* Sp.	4.2 ± 0.45	8.4 ± 0.55	21.3 ± 0.3	−−
5.	*P. aeruginosa*	6.3 ± 0.35	10.1 ± 0.41	16.8 ± 0.2	−−
6.	*K. pneumoniae*	5.2 ± 0.4	11.5 ± 0.32	17.7 ± 0.45	−−

mm: millimeter; C: control; −− no inhibition.

**Table 5:** Antifungal activity of CE@ZIF-8.

S. No	Name of the Strains		Zone of Inhibition (mm)		
		100 μg/mL	200 μg/mL	(+) ve C	(−) ve C
1.	*C. krusei*	3.2 ± 0.36	7.2 ± 0.15	10.2 ± 0.41	−−
2.	*C. propicalis*	3.3 ± 0.45	5.1 ± 0.25	14.4 ± 0.3	−−
3.	*C. albicans*	3.1 ± 0.35	6.3 ± 0.32	12.1 ± 0.4	−−

mm: millimeter; C: control; −− no inhibition.

**Table 6:** Percentage of MDA-MB-231 cell viability after treatment with different concentrations of CE@ZIF-8.

Concentration	% of Cell Viability
0 (NC)	100
15 µg/mL	90.6 ± 1.26
30 µg/mL	79.6 ±1.42
45 µg/mL	65.4 ± 1.11
60 µg/mL	53.3 ±1.30
75 µg/mL	41.4 ± 1.20
90 µg/mL	29.9 ± 1.27
105 µg/mL	17.1 ± 1.55
120 µg/mL	5.6 ± 1.63

## Data Availability

The original contributions presented in the study are included in the article.
